# Limitations of Traditional Models for Medical Decision-Making Capacity and Ethical Clinical Practice in Light of the SARS-CoV-2 Pandemic

**DOI:** 10.7759/cureus.14716

**Published:** 2021-04-27

**Authors:** Kenneth C Novoa, Thom Dunn, Ashley Curry, Richard Froude, Scott A Simpson

**Affiliations:** 1 Psychiatry, Denver Health Medical Center, Denver, USA; 2 Psychiatry, University of Colorado School of Medicine, Denver, USA

**Keywords:** ethics, capacity, covid-19, sars-cov-2, consultation-liaison psychiatry

## Abstract

The coronavirus disease 2019 (COVID-19) pandemic has upended psychiatric practice and poses unprecedented challenges for maintaining access to quality care. We discuss the ethical challenges of treating a patient with schizophrenia in need of hospitalization but who declined severe acute respiratory syndrome coronavirus 2 (SARS-CoV-2) surveillance testing. The traditional framework of capacity assessment depends on the patient’s ability to weigh risks and benefits, but this framework is of limited utility in context of the COVID-19 pandemic; the personal benefits of testing for the patient are unclear and in fact may not outweigh the risk of being declined psychiatric care. Moreover, classic capacity assessment does not well account for physicians’ obligations to other patients and the public health. We conclude that physicians cannot coerce surveillance testing, and we consider the implications of requiring SARS-CoV-2 testing for accessing mental health treatment.

## Introduction

The extensive use of behavioral interventions to reduce the transmission of severe acute respiratory syndrome coronavirus 2 (SARS-CoV-2) has highlighted the role of individual responsibility in protecting public health. Private, local, state, and federal guidelines to reduce viral transmission have prompted public discussion about how to best proceed without impingement on civil liberties [1]. When an individual is admitted to a general hospital, the patient-doctor relationship comes to include additional layers of complexity when determining physicians’ responsibility in protecting the public’s health, given the ethical responsibilities the physician has with not only the individual patient but also society at large. Below, we examine a case where a physically asymptomatic patient with schizophrenia declined SARS-CoV-2 surveillance testing throughout admission to an inpatient psychiatric unit.

## Case presentation

“Mr. D” is a 42-year-old man, presently living in homeless shelters, with an unknown psychiatric history who was in custody at the city jail for three weeks on a trespassing charge. While in jail, Mr. D demonstrated disorganized behavior and thinking as well as paranoid delusions. He refused most food during his incarceration due to his belief that the food was poisoned. He also refused all recommended psychiatric medications. Upon release he was placed on a 72-hour civil involuntary treatment order and sent to the local psychiatric emergency service (PES) for further evaluation. In the PES, Mr. D explained that he had been in jail as a volunteer to improve the facility and that the food there was poisoned. No prior treatment records were available at the time of admission. The PES psychiatrist diagnosed an acute exacerbation of schizophrenia and continued the involuntary treatment order due to evidence of grave disability. The patient was admitted to inpatient psychiatry for further treatment.

The same day, the hospital had instituted a policy requiring all medical and psychiatric admissions to receive a surveillance screening test for the novel coronavirus (SARS-CoV-2) regardless of symptom status. While in the PES, Mr. D declined the required nasopharyngeal swab. He stated that it was “against my beliefs to give bodily fluids like saliva, blood, semen, sweat, so on” and that any such fluid was “a child of mine.” He similarly declined all laboratory testing. His vital signs were unremarkable, and he had no signs or physical symptoms of coronavirus disease 2019 (COVID-19). After discussion with department leadership, the decision was made to proceed with inpatient psychiatric admission despite the patient’s refusal of testing.

On admission Mr. D continued to endorse delusions. His thinking was disorganized and his thought process loose. He was found to be responding to internal stimuli and continued to refuse all psychiatric medications. On hospital day two, he reported diarrhea. Mr. D again declined SARS-CoV-2 testing for fear that the specimen would be used to clone him. He was unfamiliar with coronavirus or the disease. He suggested that “coronavirus has to do with the coronary and the heart.” No surrogate decision-maker was available. Mr. D remained calm and without agitation throughout his admission.

At the conclusion of the 72-hour involuntary treatment order, Mr. D was felt to not meet statutory criteria for further involuntary hospitalization or for involuntary medication administration. He was referred to a local community mental health clinic and discharged without incident in accordance with his wishes. Records from the state hospital arrived after discharge, which revealed that the patient had a 20-year history of psychosis and a myriad of psychiatric hospitalizations. The case was subsequently reviewed by departmental leadership and a member of the hospital bioethics committee. Since Mr. D’s hospitalization, there have been additional cases of patients refusing surveillance screening for SARS-CoV-2.

## Discussion

This case raises several questions about how to proceed with universal SARS-CoV-2 testing in the time of a pandemic, particularly in patients who are refusing said testing. Below, we will examine the case through the lens of various ethical principles.

Autonomy, testing, and the capacity paradigm

Clinicians respect patients’ autonomy by allowing them to make informed and voluntary decisions when it comes to their treatment. The patient’s capacity for medical decision-making requires an ability to manipulate the clinical information presented and make consistent informed decisions [2]. Patients with schizophrenia often retain the capacity to make many decisions regarding their care, though their ability to do so may be impaired during the acute phase of illness [3]. These patients may require a surrogate decision maker to represent the patient’s wishes [4].

Under the traditional assessment of medical decision-making, Mr. D does not have the capacity to refuse a SARS-CoV-2 test. However, any consideration of capacity must consider the potential risks and benefits for the patient. Higher risk scenarios require more scrutiny of the patient’s decision-making capacity. In this instance of surveillance testing for coronavirus, the risk-benefit analysis is difficult to apply.

Risks and benefits, and for whom?

The benefits of surveillance screening are unclear, both for the inpatient psychiatric unit as well as for Mr. D personally. The role of universal screening prior to hospital admission remains uncertain. There is no current recommendation for such screening from the Centers for Disease Control and Prevention (CDC), which defers to clinicians to “use their judgment to determine if a patient has signs and symptoms compatible with COVID-19 and whether the patient should be tested” [5]. Arguments against universal testing include the test’s poor sensitivity and limited testing supplies [6]. The potential benefit of universal screening would be to identify asymptomatic carriers in an effort to reduce transmission [7]. If he were positive, Mr. D would indeed be at risk for transmitting SARS-CoV-2 because inpatient psychiatric units pose a particular challenge for infection control given their high density, compact space, frequently shared surfaces, and patients who have difficulty adhering to infection control guidelines [8].

For Mr. D personally, the benefits of surveillance screening are even less clear. True, he is at an elevated risk for carrying SARS-CoV-2. Higher risk populations include the homeless, the incarcerated, and those with chronic mental illness. These higher rates of infection are due to challenges in practicing physical distancing, optimum personal hygiene, and other protective measures [9,10]. Diarrhea reported by Mr. D after admission may have represented an early symptom of COVID-19 [11]. Mr. D also carries a personal interest in the resolution of the pandemic. One could speculate the positive down-stream consequences of surveillance testing for Mr. D. If he were admitted to the psychiatric unit without testing, was infected with the virus, and spread the infection to vulnerable patients who subsequently died, would Mr. D, upon successful treatment of psychosis, be upset to learn that he played a role in the death of other patients? Would he be victim to further stigmatization that could potentially increase his risk for suicide in the future? Would he take legal action against the physician and hospital for not protecting himself and others from this outcome during a time when he was incapable of making informed medical decisions? Conducting surveillance testing in this case would be seen as being of great benefit to Mr. D and those around him. However, we argue that a positive surveillance test would have significantly and negatively impacted Mr. D’s psychiatric care. He would have been transferred to a medical unit, with superior infection control measures, but he would then have been denied the therapeutic milieu of the inpatient psychiatry unit. As there is no indicated medical treatment for his asymptomatic infection, the direct benefits to Mr. D of such a transfer are minimal in the acute setting. Furthermore, the CDC recognizes personal stigmatization associated with a positive test result [12]. Mr. D himself would also be liable for the costs of testing. Ultimately, there is no direct clinical benefit to Mr. D receiving the test and perhaps only substantial harms.

Mr. D thus shoulders the entirety of the risk of testing while standing to gain only minor personal benefit. Under such circumstances, the classic decision-making paradigm allows Mr. D to refuse surveillance testing or at least limits his physicians from compelling the test.

Beneficence, nonmaleficence, and the physician’s role

Clinicians hold a duty to act in the interest of their patients, described as the ethical principle of beneficence. Mr. D’s case demonstrates the conflict between the application of beneficence to the individual patient versus society as a whole. Given the unequal burden of risk in this case, the physician is forced to consider how to prioritize competing interests between the patient and society. The role of dual stewardship has been described as physicians’ simultaneous obligation to both individual patient care and to the functioning of the larger health system [13]. This stewardship may extend to appropriate use of limited testing supplies as well when considering physicians’ obligations to society. In a global pandemic, should the physician determine that the benefits to society outweigh the benefits to the individual patient?

Such utilitarian decision-making is limited by the aforementioned uncertainty over the benefits of testing. Certainly, a positive test would represent a chance for reducing transmission risk. There are numerous examples in psychiatry and medicine where the principle of beneficence is prioritized over patient autonomy: involuntary treatment due to danger to others, the duty to warn, or directly observed treatment of tuberculosis patients. However, these examples may hold limited precedent. In the case of most involuntary psychiatric treatments, the physician’s role is justified by the intent to improve the patient’s condition via treatment rather than solely out of a need to mitigate dangerousness [4]. These impingements on autonomy are also accompanied by well-defined laws and processes for protecting patients’ rights. No such framework exists for compelling surveillance coronavirus testing.

We also consider the principle of nonmaleficence, by which clinicians must ensure that the patient is not harmed or injured through acts of commission or omission. Setting aside the discomfort and minimal physical risks of a nasal swab, the administration of such a test in an unwilling participant would necessitate the use of coercive restraints and perhaps medications. These interventions may be traumatizing for the patient and may also pose risk of injury for staff [14].

The principle of nonmaleficence also extends to the clinician’s other psychiatric inpatients who may be put at risk by the psychiatrist and patient’s decision. Other patients will not be aware of Mr. D’s refusal; psychiatric inpatients are not given the opportunity to decide for themselves the potential risk of remaining hospitalized. For some of these patients, the benefits of psychiatric hospitalization may not be outweighed by the risk of exposure to a carrier of SARS-CoV-2; the psychiatrist alone will carry the responsibility for determining that risk. Patients who are least cognizant of these potential risks may be exactly those who are at most significant risk for complications or further transmission of SARS-CoV-2 given that severe persistent mental illness is associated with medical comorbidity, homelessness, and incarceration. For involuntary patients who cannot leave, the care team carries the responsibility for ensuring safe treatment and recognizing the potential risk for infection in the inpatient unit. Mr. D’s decision to forego testing jeopardizes others’ autonomy of decision-making, the benefits of psychiatric admission for vulnerable patients, and the contributions of the inpatient psychiatric unit among the community’s continuum of care.

Ultimately, the decision to test Mr. D invokes the concept of double effect: whether an intrinsically good act is permissible if it also produces a bad or undesirable effect [15]. While physicians may feel a moral obligation to test for SARS-CoV-2 in order to limit the spread of the virus, the bad effect of restraining Mr. D and compelling the test does not outweigh the potential benefit to Mr. D and other psychiatric inpatients.

## Conclusions

This case demonstrates the limitations of traditional models for medical decision-making capacity and ethical clinical practice in light of the novel coronavirus pandemic. Evolving clinical practices, including those around surveillance screening, will continue to challenge the roles of the physician and patient in decision-making. Our hospital has subsequently seen additional cases of testing refusal, suggesting this scenario will grow more common. In the case of Mr. D, the physician’s clinical and ethical responsibilities to the patient aligned with the patient’s decision to decline testing. The physician’s professional relationship with the patient remains paramount and codified by legal and social expectations. This relationship is more certain than the less clear responsibility of Mr. D’s physician to the larger public to reduce the risk of transmission. The ethical arguments for mandatory screening cannot come at the expense of an individual’s autonomy, and the physician’s clinical judgment and expertise determine the appropriateness of testing.

Ethical expectations to mandate screening will inevitably jeopardize individual rights, particularly of vulnerable psychiatric patients like Mr. D. Striking the balance between individual rights and public health requires informed political adjudication that incorporates these competing interests into a legal framework that provides due process for patients, legal protection for clinicians, and proven benefits for public health.
